# Personalized Guidance of Edge-to-Edge Transcatheter Tricuspid Valve Repair by Multimodality Imaging

**DOI:** 10.3390/jcm13102833

**Published:** 2024-05-11

**Authors:** Alexandru Patrascu, Donat Binder, Ibrahim Alashkar, Peter Schnabel, Wilfried Stähle, Osama Risha, Kai Weinmann, Ilka Ott

**Affiliations:** 1 Department of Cardiology, Rhythmology, Electrophysiology and Angiology, Helios Hospital Pforzheim, 75175 Pforzheim, Germany; donat.binder@helios-gesundheit.de (D.B.); ibrahim.alashkar@helios-gesundheit.de (I.A.); peter.schnabel@helios-gesundheit.de (P.S.); wilfried.staehle@helios-gesundheit.de (W.S.); osama.risha@helios-gesundheit.de (O.R.); kai.weinmann@helios-gesundheit.de (K.W.); ilka.ott@helios-gesundheit.de (I.O.); 2 Faculty of Medicine, Private University in the Principality of Liechtenstein (UFL), 9495 Triesen, Liechtenstein

**Keywords:** tricuspid regurgitation, transcatheter tricuspid valve repair, edge-to-edge repair

## Abstract

**Background**: Transcatheter edge-to-edge tricuspid valve repair (T-TEER) for tricuspid regurgitation (TR) is always guided by transesophageal echocardiography (TEE). As each patient has unique anatomy and acoustic window, adding transthoracic echocardiography (TTE) and cardiac CT could improve procedural planning and guidance. **Objectives**: We aimed to assess T-TEER success and outcomes of a personalized guidance approach, based on multimodality imaging (MMI) of patient-tailored four right-sided chamber views (four-right-ch), as depicted by CT, TTE, TEE and fluoroscopy. **Methods**: Patients were assigned to MMI or classical TEE guidance, depending on TTE acoustic window. In MMI patients, planning included cardiac CT, which determined the fluoroscopic angulations of the specific four-right-ch, while guidance relied heavily on TTE, with minimal intermittent TEE for leaflet grasping and result confirmation. Both TTE and TEE were matched to respective CT and fluoroscopy four-right-ch. TR severity and quality of life (QoL) parameters were assessed from baseline to 12 months. **Results**: A total of 40 T-TEER patients were included, with 17 procedures guided by MMI and 23 solely by TEE. Baseline characteristics were similar between groups, e.g., age (83.1 ± 4.1 vs. 81 ± 5.3, *p* = 0.182) or STS-Score (11.1 ± 7.4% vs. 10.6 ± 5.9%, *p* = 0.813). The primary efficacy endpoint of ≥one-grade TR reduction at 30 days was 94% (16/17) in MMI vs. 91% (21/23) in TEE patients, with two or more TR grade reduction in 65% vs. 52% (*p* = 0.793). Device success was overall 100%, with no device-related complications, but three TEE-associated cases of gastrointestinal bleeding in the TEE-only group. By 12 months, all 15 MMI and 19 TEE survivors improved NYHA functional class and QoL, e.g., Kansas City Cardiomyopathy Questionnaire Score Δ29.6 ± 6.7 vs. 21.9 ± 5.8 (*p* = 0.441) pts., 6-min walk distance Δ101.5 ± 36.4 vs. 85.7 ± 32.1 (*p* = 0.541) meters. **Conclusions**: In a subset of patients with good TTE acoustic window, MMI guidance of T-TEER is effective and seems to avoid gastroesophageal injuries caused by TEE probe manipulation. TR reduction, irrespective of guidance method, impacts long-term QoL.

## 1. Introduction

### 1.1. Status Quo

High-grade tricuspid regurgitation (TR) is increasingly prevalent nowadays [1], owing to an aging population with ever more comorbidities [2], and caries a dismal prognosis without proper treatment. As tricuspid valve (TV) surgery for isolated TR is rarely performed in the elderly, owing to high postsurgical mortality [3], and considering that medical therapy only contributes to symptom control, most symptomatic patients are not offered any specific treatment [4]. Recently, transcatheter edge-to-edge tricuspid valve repair (T-TEER) proved to be safe and effective in inoperable patients [5], and it is even mentioned in current guidelines [6].

As was the case in the beginning with transcatheter aortic valve replacement and is still the case with transcatheter edge-to-edge mitral valve repair (M-TEER) [7], transesophageal echocardiography (TEE) plays a crucial role in T-TEER guidance, in conjunction with fluoroscopy.

### 1.2. Why the Need for a Personalized Guidance Approach

As creatures of habit, it is understandable to use the proven TEE-only guidance of M-TEER and extrapolate this approach to T-TEER. However, while mitral and tricuspid valve devices may be very similar, anatomy and procedural steps are not alike.

First, from an anatomical point of view, the tricuspid valve lies more anterior and apical than the mitral valve, and can, in theory, be better visualized with transthoracic echocardiography (TTE). Provided good acoustic window, accurate TV leaflet identification is often possible on TTE, using a comprehensive two-dimensional and multiplanar interrogation [8,9,10]. Thus, TTE could be very valuable during T-TEER, not only for screening and follow-up investigations. In comparison, considering the anterior position of the TV, TEE images are prone to shadowing artifacts by more posterior structures closer to the probe.

Second, while TEE is regarded as very low-risk, procedural guidance relies heavily on a constant alternation between mid-, deep esophageal and transgastric views. However, a recent study on TEE safety during T-TEER [11] has shown a rate of periprocedural esophageal and gastric injuries of 60%.

Third, from an interventional point of view, there is little common ground between fluoroscopy and echo. In order to mend both, cardiac CT might build a bridge between the operator and the cardiac imaging specialist, as it can predetermine fluoroscopic angulations. Recently, the concept of the four right-sided chamber views was introduced, which takes into account the fluoroscopic position of the TV annular plane in relation to the right-sided heart chambers [12,13], as reconstructed by CT imaging.

### 1.3. Aim of the Study

Therefore, the current “one size fits all” approach to T-TEER guidance, by using solely TEE, mostly independent from fluoroscopy, seems worthy of improvement, especially by adding TTE and cardiac CT. Hence, we tested the hypothesis that T-TEER can be successfully conducted by a personalized multimodality imaging (MMI) approach, tailored to individual anatomy, based on the concept of the four right-sided chamber views.

## 2. Materials and Methods

### 2.1. Study Design and Patient Selection

Forty consecutive patients undergoing T-TEER at our institution between December 2020 and January 2022 were prospectively included in the Pforzheim Tricuspid Valve Registry (NCT05179616), after giving written informed consent. Patients were highly symptomatic and deemed ineligible for conventional surgery by the Heart Team. Only secondary functional TR of either atrial or ventricular cause, or both, was treated. Patients with primary and cardiac implantable device-related TR were excluded. Other important exclusion criteria were treatable left heart disease, e.g., high-grade mitral regurgitation, and severe pulmonary hypertension, defined by an invasive systolic pulmonary artery pressure >70 mmHg.

The cohort was divided into two groups (Table 1), according to the respective T-TEER guidance approach: either classical TEE guidance, or personalized MMI guidance. Allocation to the MMI group was primarily based on very good visualization of TV leaflets in all four right-sided chamber views on TTE, confirmed in supine position, but also on information gained from cardiac CT (see imaging protocol).

Follow-up investigations were conducted at discharge, 30 days, 6 and 12 months. The analysis was approved by the local ethics committee (state medical association of Baden-Wuerttemberg, Stuttgart, Germany).

### 2.2. Study Endpoints

The primary clinical endpoint was a reduction in TR severity of at least one grade between baseline and 30-day follow-up, which also represented procedural success, in line with the approval study for the lone CE-marked T-TEER device at the start of this research project in 2020 [14]. The primary non-clinical endpoint was defined by safe clip placement, which marked device success. Secondary endpoints related to improvement in quality of life at 12 months and parameters of right heart reverse remodeling. The safety endpoint was a composite of major adverse events (Tricuspid Valve Academic Research Consortium) [15].

### 2.3. Studied Variables

Examined parameters can be classified into several categories: baseline characteristics (e.g., demographic data, surgical risk and medical history), procedural data (e.g., technical details and radiation doses), clinical outcomes [New York Heart Association (NYHA) functional class, Kansas City Cardiomyopathy Questionnaire Score (KCCQ) and 6-min walk test], laboratory values for renal and hepatic function, and echocardiographic parameters of right heart remodeling.

### 2.4. Imaging Protocol

**Figure 1:**
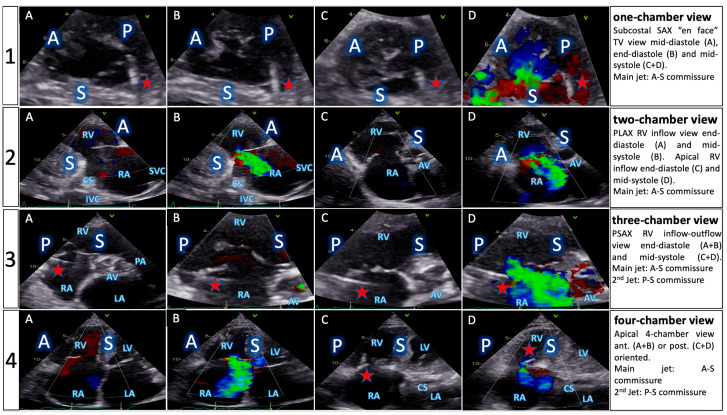
TTE screening protocol. Comprehensive evaluation of TV anatomy based on the concept of the four right-sided chamber views (patient with massive TR). A: anterior leaflet, P: posterior leaflet, S: septal leaflet, RV: right ventricle, RA: right atrium, CS: coronary sinus, IVC: inferior vena cava, SVC: superior vena cava, AV: aortic valve, PA: pulmonary artery, LV: left ventricle, LA: left atrium, red star: pacemaker lead.

All patients underwent complete preprocedural screening consisting of TTE (Figure 1, Table 2) and TEE (Supplementary Figure S1), before they were assigned to one of the groups. MMI patients also received cardiac CT.

Further TTE investigations were performed at discharge and during follow-up visits (Supplementary Figure S2). Echocardiograms followed current ASE and EACVI [16] guidelines. All investigations were assessed independently by three cardiac imaging specialists blinded to procedural details. TR severity was graded using the five-grade scheme [17].

**Figure 2:**
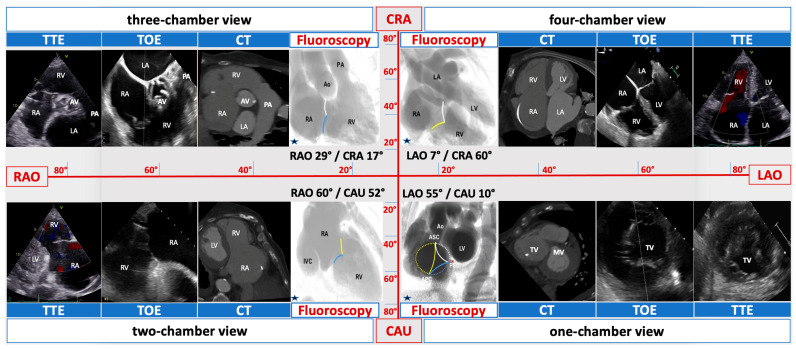
Multimodality imaging of the right heart. Matching echocardiographic transesophageal (TEE) and transthoracic (TTE), CT and fluoroscopic views of right-sided heart structures in a patient scheduled for T-TEER. RV—right ventricle, RA—right atrium, TV—tricuspid valve, MV—mitral valve, AV–aortic valve, PA-pulmonary artery, LV—left ventricle, LA—left atrium, CAU—caudal, CRA—cranial, LAO–left anterior oblique, RAO—right anterior oblique, blue star: reproduction of angiographic images with permission from Elsevier/JACC [13].

In MMI patients, T-TEER was guided by a carefully planned combination of echocardiography, mostly TTE (see procedural protocol below), and fluoroscopy, based on understanding of TV anatomy, in relation to the right-sided chamber views. For consistency reasons, cardiac structures are described in line with their attitudinal position in the body, regardless of imaging method. The starting point was visualizing the TV annular plane in the four right-sided chamber views (Figure 2) by echocardiography and matching these views to predetermined fluoroscopic angulations.

Accordingly, the one-chamber view, usually seen in the transgastric short-axis TV view on TEE, was acquired on TTE from a modified subcostal short axis [18] (Videos S1 and S2). More precisely, the transducer was turned 90° counterclockwise from the usual subcostal long axis, so the posterior leaflet was always on the right-hand side of the image, the anterior on the left-hand side, and the septal pointing toward the bottom of the screen, respectively, toward the mitral valve. Alternatively, in some patients, the also called “en face” TV view was obtained either from a modified parasternal short-axis view upon gentle tilting of the probe towards the right shoulder, or from using 3D images.

Next, the two-chamber view, or right ventricular (RV) inflow view, normally visualized on TEE from a low-esophageal or transgastric position, was acquired on TTE mainly from a parasternal long axis view of the right heart, alternatively using a standard apical 2-chamber view, with the probe tilted toward the right chambers. Important for T-TEER planning, TTE distinction between TV leaflets was possible in most cases, with the anterior leaflet on the right side of the image and either the septal or posterior leaflet on the left side, depending on whether the interventricular septum was within plane or not, during gentle probe tilting. In direct comparison to the two-chamber deep esophageal TEE view, it is safe to say that the TTE parasternal long axis view of the RV was always of better quality, and more accurately depicted the “true” RV inflow view.

Furthermore, the three-chamber view, corresponding to the RV inflow-outflow view, typically seen on mid-esophageal TEE 50–100°, was obtained using the standard parasternal short-axis view on TTE. Leaflet distinction on this short axis was sometimes aided by 3D imaging, but distinguishing between the posterior and anterior leaflets was not always obvious without 3D acquisitions. As for the more medial positioned leaflet in the image, concomitant visualization of the aortic valve in 2D pointed toward the anterior leaflet, while the presence of the interventricular septum toward the septal one.

Finally, the four-chamber view, seen on mid-esophageal 0–30° TEE, corresponded to the classical four-chamber view on TTE. Thereby, the septal leaflet was always the one closest to the septum, while distinguishing between the anterior and posterior leaflets was facilitated by the presence of either the left ventricular outflow tract (anterior leaflet) or coronary sinus (posterior leaflet).

Biplane imaging and 3D acquisitions were performed routinely during screening, made most views interchangeable, and helped with leaflet identification, especially using multiplanar reconstruction (MPR).

### 2.5. Procedural Protocol

T-TEER was performed exclusively with the TriClip^TM^ (Abbott Medical, Tokyo, Japan) device [14], using XT and XTW clips, in accordance with institutional guidelines. The team consisted of three interventional cardiologists, two cardiac imaging specialists, and a rotating anesthesiologist, all with substantial M-TEER experience.

All patients received general anesthesia, with TEE probe placement immediately after endotracheal intubation in the TEE guidance group (Supplementary Table S1). In the MMI group, however, the procedure was guided mostly by aforementioned TTE 2D and biplane views, with intermittent probe intubation and TEE use only before clip grasping and release, to check for sufficient leaflet insertion and confirm the result, or in case of imaging ambiguities. This kept the TEE time to a minimum and, in most cases, avoided probe passage through the gastroesophageal junction. Of note, 3D/MPR views from parasternal and apical perspectives were affected by invasive ventilation and were rarely employed periprocedurally. At the same time, their main value was in leaflet identification during screening, and did not seem to offer more information than 2D and biplane acquisitions. Nonetheless, the one-chamber subcostal view was mostly unaffected by invasive ventilation. Considering the longer distance from the probe to the right heart in subcostal views, 3D images were again not useful, but, at the same time, also not necessary when directing the clip toward the commissure using 2D and biplane imaging. In comparison, in the TEE-only group, except for two cases, acoustic 3D-window was insufficient to guide the procedure, so classical 2D/biplane acquisitions, especially transgastric, were mandatory.

Imaging and fluoroscopy were synchronized to the tune of the predetermined four right-sided chamber views in the MMI group, as soon as the steerable guide catheter was advanced into the right atrium (RA), under fluoroscopic guidance. More precisely, during aforementioned TTE screening in supine position, the best possible four right heart chamber views were marked and then searched for during reconstruction of 3D-images from CT acquisitions, by paying attention to different anatomical structures. For example, when determining the best two-chamber view in the parasternal long axis of the RV, if the inferior vena cava was located at 7 o’clock and the coronary sinus at 8 o’clock, these exact positions were searched for during CT reconstruction. This was possible with a dedicated software (Horos^TM^, Horos Project, Version 3.3.6) that allowed integration of both TTE and CT images. Finally, after software assisted matching, the expected CT-derived C-arm angulations were noted, in a similar manner to TAVR procedures.

Therefore, clip advancement inside the RA was performed under both fluoroscopy and echocardiography, more or less simultaneously in the TEE group, or in alternation in MMI patients. This was imperative in order to avoid exposing the imaging specialist to unnecessary radiation, as TTE images were acquired from the patients left side. Moreover, the imager had a movable radiation shield in front, and kept about the same distance to the source of radiation as the interventionalist. The echo machine was also positioned on the left side of the patient, unlike the TEE-only approach, where it was located behind the head of the patient, also separated by a protection shield. Overall, we measured the same distances between the echo machine, radiation screen, imager and patient in both groups, with the single difference in the MMI group of having the imager’s hand reach out behind the protection screen, which is why there was a need for alternation of fluoroscopy and echo.

**Figure 3:**
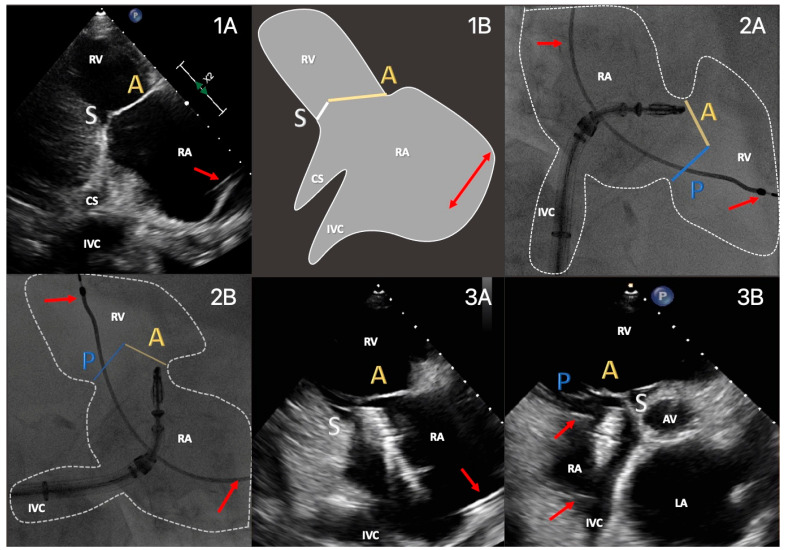
Clip advancement in the two-chamber transthoracic view. Advancement of clip delivery system from the inferior vena cava (IVC) into the right atrium (RA) using corresponding transthoracic and fluoroscopic views. (**1A**): right ventricular parasternal long axis, (**1B**): schematic illustration, (**2A**): anterior-posterior fluoroscopic projection, (**2B**): matching 3D rotation of the TTE view, (**3A**,**3B**): multiplanar two- and three-chamber views directing the clip toward the valve. A/P/S: anterior, posterior or septal leaflet, RV: right ventricle, CS: coronary sinus, IVC: inferior vena cava, AV: aortic valve, LA: left atrium, red arrow: pacemaker lead (posterior course).

The first step in the MMI approach was pointing the clip toward the TV, which on TEE is easily guidable using bicaval and short-axis midesophageal views. On TTE, however, this is also possible using the parasternal long axis RV inflow view, which contains both caval veins (Figure 3), aided by the parasternal short axis, through biplane imaging. More specifically, as soon as the clip delivery system entered the acoustic TTE window, mostly on the right-hand side of the parasternal RV inflow view, coming from the superior vena cava toward the inferior one, the C-arm was rotated to a right anterior oblique angulation and CT-aided predetermined matching TTE/fluoroscopic views allowed perpendicular arrangement and advancement of the clip toward the TV. Thus, slow clockwise rotation of the guide catheter directed the clip toward the TV, while four- and mainly two-chamber TTE views of the right heart allowed clip visualization. Intubation of the coronary sinus was avoided both on fluoroscopy and on two-/four-chamber views.

**Figure 4:**
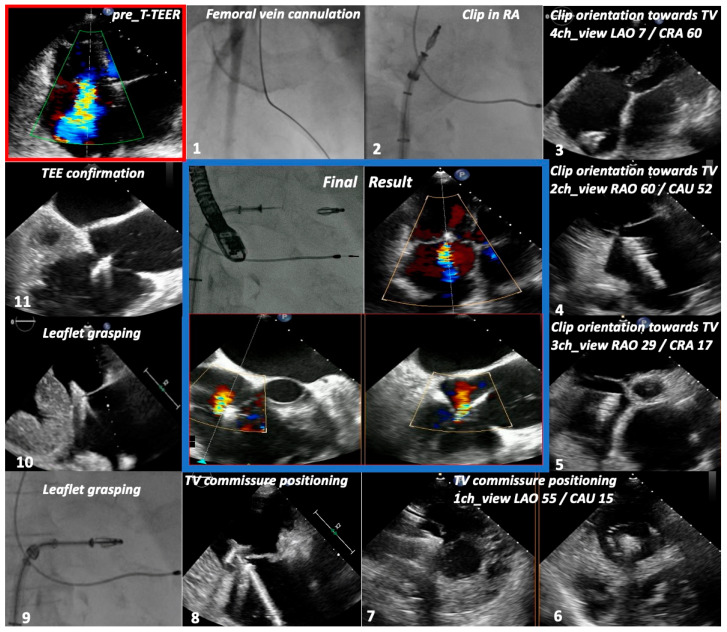
Procedural steps of MMI-guided T-TEER. Sequence of personalized multimodality imaging guidance of T-TEER using the four right-sided chamber views, tailored to the individual anatomy of the patient from Figure 2. TR: tricuspid regurgitation, CDS: clip delivery system, RA: right atrium, TV: tricuspid valve, ch: chamber.

Upon reaching valve proximity, clip orientation toward the desired commissure was facilitated by the “en face” one-chamber view, respectively, left anterior oblique caudal fluoroscopic angulation. Fine-tuning along the chosen commissure was mostly performed using two- and three-chamber views, corresponding on fluoroscopy to the right anterior oblique caudal and cranial projections (Figure 2). At this point in time, TEE was employed in order to confirm both clip position before grasping, and the final result. Finally, leaflet grasping was performed using the two- and four-chamber TTE views, matching the right anterior oblique caudal angulation and, respectively, the cranial left anterior oblique. The periprocedural value of the four right-sided chamber views can be appreciated in Figure 4, Supplementary Figure S3, and Video S3.

Of note, in cases where two or more clips were needed, two main strategies to reduce TR were employed, either the clover or the bicuspidalization technique. The clover method aimed at preserving three orifices by placing the clips centrally between the septal and anterior, as well as between the septal and posterior leaflets, respectively. This technique made more sense in patients with pacemaker leads, so not to pinch the lead. More often, bicuspidalization was used by placing two or more clips in the anteroseptal commissure, mainly because most patients had large coaptation defects. Clip implantation at the level of the anteroposterior commissure or between scallops of the posterior leaflet was avoided.

It is worth mentioning that some CT-derived fluoroscopic viewing angles were not always easy to achieve on the C-arm (e.g., RAO 60°, CAU 60°), so compromise was needed for practical reasons. Also, the presence of pacemaker leads proved to be both beneficial and detrimental. The main advantage with MMI guidance was always knowing where the corresponding leaflet or commissure was located, while the obvious disadvantage arose from an interventional point of view, as the grasping attempts needed to take into account lead position.

### 2.6. Statistical Analysis

Normal distribution was first confirmed by using the Shapiro-Wilks test. Two main sets of variables were identified: continuous and categorical parameters. The former set is expressed as mean ± SD, while the latter is presented as frequencies and percentages. Statistical comparisons were performed either within a group, e.g., change in a clinical parameter following T-TEER, or between groups, e.g., change in an echocardiographic variable between MMI and TEE groups. The paired Student’s *t*-test and the Wilcoxon’s signed-rank were used for in-group analyses, while the McNemar’s test served the few nominal variables. Between-group comparisons were realized with either the independent Student’s *t*-test or the Fisher’s exact test. Statistical significance was defined as *p* < 0.05, as calculated using two-tailed tests. Paired analyses for all baseline characteristics are provided in Supplementary Table S3. Statistical analyses were performed using SPSS version 26 (IBM, Armonk, NY, USA). The first author had full access to all the data in the study and takes responsibility for its integrity and the data analysis.

## 3. Results

### 3.1. Study Population

Seventeen patients (42.5%) were assigned to the MMI group, while twenty-three (57.5%) underwent classical TEE guiding (Table 1, Supplementary Table S3).

In accordance with the proposed guiding approach and subgroup definition, a statistically significant difference in transthoracic image quality was present, with all MMI patients having excellent TTE acoustic window. Of note, within the time frame of this study, 124 patients with either TR as the single manifestation of valvular heart disease, or with both mitral and tricuspid regurgitation, were screened for TTE quality of the aforementioned four right-sided chamber views, before discussing each individual case with the Heart Team. Thirty-eight patients (31%) were considered to have excellent TTE acoustic window and to possibly qualify for the MMI approach. While only seventeen were eventually included, the rest had either TR improvement after MR reduction, refused T-TEER after M-TEER despite persistent severe TR, or were recommended surgical therapy by the Heart Team.

With the exception of body mass index (BMI) 22.9 ± 1.1 vs. 30.4 ± 3.7 (*p* < 0.001), there was no obvious between-group difference in standard baseline parameters like age (83.1 ± 4.1 vs. 81 ± 5.3, *p* = 0.182), sex [(10/17 (59%) vs. 10/23 (44%) women, *p* = 0.595)] or surgical risk scores [EuroSCORE II 10.1 ± 8.2 vs. 8.6 ± 5.6%, *p* = 0.496; Society of Thoracic Surgeons Score (STS) 11.1 ± 7.4 vs. 10.6 ± 5.9%, *p* = 0.813]. Patients had similar comorbidities, which included major organ system compromise (2.5 ± 1.4 vs. 2.3 ± 1.1, *p* = 0.616), e.g., chronic kidney disease stage 3 or worse [(14/17 (82%) vs. 19/23 (83%), *p* = 1.000], cardiovascular risk factors, e.g., type 2 diabetes [(6/17 (35%) vs. 10/23 (43%), *p* = 0.773], and prior cardiac disease, e.g., coronary artery disease [(8/17 (47%) vs. 15/23 (65%), *p* = 0.339]. Likewise, no obvious difference in symptom burden and quality of life was recorded, whether NYHA functional class III–IV [15/17 (88%) vs. 20/23 (87%), *p* = 1.000], KCCQ Score (32.3 ± 18.7 vs. 26.7 ± 14.7 points, *p* = 0.315) or 6-min walk distance (183.2 ± 91.4 vs. 162.1 ± 94.1 m, *p* = 0.480).

### 3.2. Procedural Characteristics

Procedural success was achieved in all but three patients, [94% (16/17) MMI group vs. 91% (21/23) TEE group, *p* = 1.000)]. Similar between-group TR grade improvement was noticed, with most procedures achieving at least two-grade reduction [(65% (11/17) vs. 52% (12/23), *p* = 0.793] at 30 days. Thus, grade IV/V° and V/V° TR, present at baseline in 76% (13/17) of MMI and 83% (19/23) of TEE patients (*p* = 1.000), were only recorded in one MMI and two TEE cases by procedure end, and remained mostly unchanged at one and twelve months (Supplementary Figure S4).

Device success was 100%, with a total of 27 implanted clips in the MMI group (1.5 ± 0.6 clips per patient) and 34 clips in the TEE group (1.5 ± 0.5), as shown in Table 3.

Most clips were placed in the anteroseptal commissure [(52% (14/27) vs. 59% (20/34), *p* = 0.831], by a combination of three interventional techniques. In the MMI group, single clip placement was more often sufficient in TR reduction [47% (8/17)] and aimed for the main body of TR jet. True bicuspidalization was achieved in three patients by placing either two or three clips close to each other along the anteroseptal commissure, and the clover technique was performed in six cases by preserving three orifices. Overall, clip implantation led to a mild increase in TV gradients by procedure end (∆ 0.8 ± 0.6 vs. 0.8 ± 0.7 mmHg, *p* = 0.896), while right atrial pressure decreased (∆ 2.7 ± 1.3 vs. 3.2 ± 2.6 mmHg, *p* = 0.432).

Further procedural parameters like device (66.1 ± 35.1 vs. 58.7 ± 27.5 min, *p* = 0.459) and fluoroscopy time (14.4 ± 8.8 vs. 13 ± 7.1 min, *p* = 0.578) were similar between groups, with slightly higher radiation dose in the TEE group (4074.6 ± 2491.7 vs. 5125.1 ± 3827.6 cGy, *p* = 0.330), though still comparable with standard coronary angioplasty. Equally important from a radiation exposure perspective was the fact that both the interventionalist and the imager performing intraprocedural TTE received less than one microsevert (µSv) in each single procedure.

### 3.3. Safety Endpoint

All patients were discharged, and no device-related complications occurred. However, three patients in the TEE group had gastrointestinal bleeding, confirmed by gastroscopy, with one in need of blood transfusion. Of those, two were mid-esophageal and one deep esophageal, and two were due to thermal injury. By 12 months, all-cause overall mortality was 15% (6/40), with three cardiac-related deaths. The rate of hospitalization for heart failure was 0.30 events per patient-year (Table 4).

### 3.4. Secondary Endpoints

Fifteen patients in the MMI group and nineteen in the TEE group completed one-year follow-up. Improvement in parameters related to QoL and functional capacity reflected procedural success, irrespective of guidance method (Table 5).

**Figure 5:**
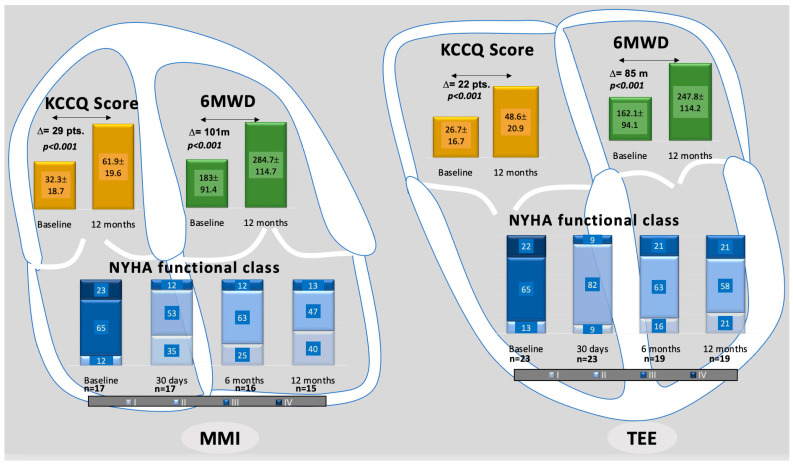
Clinical outcomes comparison. In-group improvements in functional and clinical measurements from baseline to 12 months after T-TEER. NYHA: New York Heart Association functional class. KCCQ: Kansas City Cardiomyopathy Questionnaire score (points). 6MWD: 6-min walk test distance (meter). Values are mean ± SD.

Hence, at least one-grade NYHA class improvement occurred in all survivals. Furthermore, overall KCCQ score (∆ 25.2 ± 17.7 points, *p* < 0.001) and 6-min walk distance (92.4 ± 79.1 m, *p* < 0.001) significantly increased. TR reduction also positively affected major organ systems, e.g., liver and kidney, as glomerular filtration rate increased from 53.6 ± 17.2 to 57 ± 18.7 mL/m^2^/1.73 m^2^ (*p* = 0.096), and hepatic congestion parameters decreased (e.g., aspartate aminotransferase 33.7 ± 20.7 vs. 27 ± 10.3 U/L, *p* = 0.009). For a between-group comparison, see Figure 5. Percutaneous TV repair also impacted cardiac function by reversing right heart remodeling, as assessed by echocardiography (Supplementary Table S3).

## 4. Discussion

### 4.1. Summary of Main Findings

This is the first study to explore the safety and effectiveness of a personalized guidance approach to T-TEER based on the concept of the four right-sided chamber views. The main findings are as follows:

1. T-TEER can be effectively and safely guided by a CT-aided, meticulously planned combination of TTE, intermittent TEE and fluoroscopy, tailored to the patient´s anatomy.
2. The implementation of the concept of the four right-sided chamber views builds a bridge between imaging methods involved in procedural planning and interventional guidance. Furthermore, it allows cardiac imaging specialists and interventionalists to speak a common language.
3. Irrespective of the chosen guiding method, successful TR reduction leads to improved quality of life and long-term outcomes.

### 4.2. Efficacy and Safety of the Multimodality Imaging Method

Device success of MMI-guided T-TEER (100%) and procedural success (94%) were not only comparable to classical TEE guidance, but also to published data from international registries [5,14,19]. Though not statistically significant, more-grade reduction was better in the MMI group. More importantly, the new approach proved to be safer than classical TEE guiding and avoided gastrointestinal bleeding, which is caused by either probe manipulation leading to mechanical trauma, or thermal injuries due to prolonged probe times inside the esophagus, as confirmed by gastroscopy. So, instead of repetitively running the TEE probe up and down the esophagus and stomach during the procedure, TEE was employed only intermittently during the latter critical steps. This kept the TEE usage time to a minimum, more exactly to only a few minutes, and is in our opinion the main factor behind the better safety outcome. The rate of gastrointestinal bleeding during T-TEER in the TEE group (3/23, 13%) is also in line with registry data [20].

### 4.3. Advantages and Particularities of a Personalized Approach

Screening for, and relying heavily on periprocedural TTE views, provides several benefits. First, obtaining the four right-sided chamber views leads to a comprehensive TTE understanding of TV morphology. This, in turn, enhances quality of follow-up investigations, which have a common transthoracic denominator. From our experience, when taking into consideration the distance from the tip of the transducer to the TV and the concept of the four right-sided chamber views, the distance traveled by the echo beams to the valve was often shorter in TTE than in TEE in the two- and three-chamber views (5–8 cm vs. 7–10 cm), and similar in the four-chamber view (7–11 cm). The slightly longer distance in the “en face” one-chamber view (4–6 cm subcostal vs. 3–5 cm transgastric) still offered enough information on the position of leaflets and commissures.

Second, TTE can mend the gap in case of poor TEE acoustic window, e.g., left-sided posterior structures causing shadowing in midesopageal views or hiatal hernia causing suboptimal transgastric images. We therefore strongly believe that TTE views should be incorporated into every T-TEER guidance protocol, which would fill the void in recommendations on echocardiographic guidance, as TTE is not even mentioned in recent state of the art papers [21].

Third, performing cardiac CT helps determine individual fluoroscopic angulations for the four right-sided chamber views, so that clip alignment to the TV and desired commissure can be performed also by fluoroscopy, with each specific angulation being matched to its predefined echocardiographic counterpart. The need for predetermined fluoroscopic angulations and, thus, for CT, came from the observation that standard antero-posterior views and 30 °C-arm angulations did not seem to provide a true 3D alignment to the anatomical target and were in no way coupled with echo images, so team members felt they were often working independent from one another. It is also important to mention that CT planning for T-TEER is, in theory, not mandatory, as there is very little interindividual variability of fluoroscopic angulations for right-sided heart structures [12,13]. Also, the position of the right coronary artery in relation to the TV annulus is not relevant, unlike for percutaneous procedures aimed at direct annular reduction. Nonetheless, cardiac CT before T-TEER can provide valuable information like the position and opening angle of the junction between the inferior vena cava and the right atrium, or the distance and angle between this junction and the TV annular plane. In this study, performing CT in MMI patients was crucial to establishing the personalized guidance approach and, indirectly, to achieving procedural success. Even though a general recommendation for T-TEER screening by CT cannot be made and was not our aim, we feel there is a tradeoff between the added radiation in mostly octogenarians and the morphological information obtained. With the current development of transcatheter tricuspid valve replacement, CT might become standard before tricuspid interventions and help in patient selection for replacement or repair.

Fourth, the MMI approach could shorten the learning curve for novices by exposing them to the complexity of the TV in the context of T-TEER, and it could increase their efficiency.

### 4.4. Possible Future Development

The fusion of TEE with TTE, CT and fluoroscopy is a promising development in clinical practice, which could alleviate imaging difficulties and improve procedural guidance. Even though this study compared the new MMI approach, with heavy reliance on TTE views, to classical TEE guidance, the goal was not and should not be to abandon TEE. We believe that every anatomy and procedure are unique, and careful planning, tailored to each patient, should precede T-TEER. By adding TTE and CT and building a bridge between fluoroscopy and imaging, a truly personalized approach can improve procedural guidance and results. The rapid development of live software-aided fusion imaging will certainly add further value to this concept. Of note, intracardiac echocardiography was not available for this study, as is currently the case in most countries worldwide, considering the prohibitive added costs. Also, from a distance, intracardiac echocardiography seems to fall short of providing a true one-chamber view, as found in transgastric or subcostal views. This “en face” view is in most tricuspid valves poorly reiterated by 3D images due to the usually thin leaflets.

Furthermore, one of the main inconvenient of classical TEE guidance arises from the need for general anesthesia in these high-risk TR patients. Also, dependance on availability of anesthesiologists and their surgical schedule is a limiting factor for expansion of procedural volumes, for the time being. We therefore consider the proposed MMI approach as a possible step toward conscious sedation, or an improvement in the few hospitals already experimenting with TEE-only guidance without general anesthesia, by performing guidance mainly by TTE, with intermittent use of TEE, as explained above. This does not seem far-fetched for a very safe venous procedure with no need for transseptal puncture, where the device can be visualized by a combination of three different imaging modalities at all times: fluoroscopy, TTE and TEE. Depending on specific country legislation, this could imply performing T-TEER in the absence of an anesthesiologist.

### 4.5. Clinical Outcomes

Similar to other T-TEER studies [14,19], TR reduction led to improvement in quality of life, multiorgan function and functional capacity, as well as cardiac reverse remodeling, in both groups. A statistically non-significant difference in clinical outcomes was noticed in the MMI group, which had better procedural results. As for a comparison with the lone randomized controlled T-TEER trial [5] to date, the slightly higher gain in KCCQ score of the 40 patients included in our study, as well as the considerable difference in 6-min walk distance improvement, speak to the differences in populations. The much sicker cohort of patients we enrolled (e.g., all our patients had at least two hospitalizations for acute heart failure within one year before T-TEER vs. only 25% of patients in the TRILUMINATE Pivotal trial, with one hospitalization within one year) might explain the better improvement in some parameters, as their baseline levels were also much lower, e.g., KCCQ score in our cohort 29.1 ± 17.2 vs. 56.0 ± 23.4 [5]. Moreover, considering the extremely sick population we were faced with, this study extends the knowledge gain up to one-year post- T-TEER in patients at high and mostly prohibitive risk [22].

### 4.6. Limitations

First, as with any single-arm monocentric study, local expertise is crucial and directly affects procedural success and outcomes. Implementation of the above methodology may, thus, not be generalizable. Second, the statistically low number of patients may have led to a lack of power to record significant changes in long-term outcomes. This, however, is a common problem in the early stages of T-TEER. Third, an independent echocardiography core laboratory was not available. Nonetheless, the three cardiac imaging specialists were blinded to patient data and procedural results during follow-up investigations. Fourth, not all patients with severe TR referred for interventional therapy would qualify for the MMI approach, as TTE acoustic window was considered adequate in only 31% of the individuals screened, so the cohort needs to be considered a subset of T-TEER patients.

## 5. Conclusions

Personalized guidance of T-TEER, based on multimodality imaging, is feasible and safe. Furthermore, it can offer more morphological information, improve safety, and be at least as valuable as TEE-only guidance. The concept of the four right-sided chamber views, seen from TTE, TEE, fluoroscopy and CT perspectives, creates a common language between T-TEER team members. Successful TR reduction, irrespective of guidance method, leads to significant long-term improvement in quality of life.

## Figures and Tables

**Table 1:** Baseline characteristics. Data presented as % or mean ± SD. BMI: body mass index; EuroSCORE: European System for Cardiac Operative Risk Evaluation Score; STS: Society of Thoracic Surgeons predicted risk of mortality (calculated based on isolated mitral valve replacement); NYHA: New York Heart Association; RHF: right heart failure; COPD: chronic obstructive pulmonary disease; CKD: chronic kidney disease; TIA: transient ischemic attack; ICD: implantable cardioverter defibrillator; CRT: cardiac resynchronization therapy; MV: mitral valve.

Characteristic		MMI (n = 17)	TEE (n = 23)	*p*-Value	Characteristic		MMI (n = 17)	TEE (n = 23)	*p*-Value
Clinical					Comorbidities				
Age (years)		83.1 ± 4.1	81 ± 5.3	0.182	Atrial fibrillation		16 (94%)	23 (100%)	1.000
Female sex		10 (59%)	10 (44%)	0.595	Pulmonary hypertension		16 (94%)	22 (96%)	1.000
TTE acoustic window	Excellent	17 (100%)	0 (0%)	<0.001	Type 2 diabetes		6 (35%)	10 (43%)	0.773
	Good	0 (0%)	2 (9%)	0.506	Arterial hypertension		17 (100%)	23 (100%)	1.000
	Moderate	0 (0%)	13 (57%)	0.004	COPD		3 (18%)	6 (26%)	0.719
	Poor	0 (0%)	8 (34%)	0.037	CKD stage 3–5		14 (82%)	19 (83%)	1.000
BMI (kg/m^2^)		22.9 ± 1.1	30.4 ± 3.7	<0.001	Prior stroke/TIA		3 (18%)	5 (22%)	1.000
EuroSCORE II (%)		10.1 ± 8.2	8.6 ± 5.6	0.496	Coronary artery disease		8 (47%)	15 (65%)	0.339
STS Score (%)		11.1 ± 7.4	10.6 ± 5.9	0.813	Pacemaker/ICD/CRT		5 (29%)	4 (17%)	0.712
NYHA class III–IV		15 (88%)	20 (87%)	1.000	Prior MV repair	percutaneous	7 (41%)	3 (13%)	0.164
RHF hospitalizations		2.8 ± 0.7	2.5 ± 0.7	0.188		surgical	1 (10%)	1 (4%)	1.000

**Table 2:** TTE screening protocol. PLA: parasternal long axis, PSA: parasternal short axis, A2C: apical two-chamber view, A3C: apical two-chamber view, A4C: apical four-chamber view, RH: right heart, RV: right ventricle, A4C: apical four chamber, A2C: apical two chamber, LVOT: left ventricular outflow tract, Qs: systemic flow, CO: cardiac output, TR: tricuspid regurgitation, AL: anterior leaflet, PL: posterior leaflet, SL: septal leaflet, PA: pulmonary artery, TV: tricuspid valve, PISA: proximal isovelocity surface area, RVOT: right ventricular outflow tract, VTI: velocity time integral, TAPSE: tricuspid annular plane systolic excursion, TDI: tissue doppler imaging, FAC: fractional area change, RA: right atrium.

TTE View	Focus		
	Functional Parameters	Right Heart Morphology	TV Anatomy
PLA standard	LVOT diameter (Qs/CO calculation)	RV function and size (eyeballing)	--
PLA RV inflow RH two-chamber view	TR severity (eyeballing) TR Jet VC and PISA (optional)	RV function and size (eyeballing)	AL visualization SL vs. PL distinction
PSA standard RH three-chamber view	TR severity (eyeballing) RVOT VTI RVOT diameter	RV size PA size	Leaflet distinction, if possible
PSA-modified alternative RH one-chamber view	TR severity (eyeballing)	TV annulus size Coaptation gap	Simultaneous visualization of all leaflets
A4C RH four-chamber view	TR Jet area, VC and PISA TR VTI, RVSP TAPSE RV free wall TDI RV FAC RA volume RV diameters LVOT VTI (A5C/A3C)	RV function and size RA size TV annulus size Tenting height	SL visualization AL vs. PL distinction
A2C right alternative RH two-chamber view	TR Jet area, VC and PISA	RA size TV annulus size	AL visualization
Subcostal long axis	Hepatic systolic vein flow reversal Inferior vena cava size	RV function and size (eyeballing)	PL visualization AL vs. SL distinction
Subcostal short axis RH one-chamber view	TR severity (eyeballing)	Coaptation gap	Simultaneous visualization of all leaflets

**Table 3:** Procedural characteristics. Values are % (n) or mean ± SD (n). * Successful Clip deployment and device retrieval at the end of the procedure. ** Puncture of femoral vein to access site closure *** Delivery catheter insertion to removal; TR: tricuspid regurgitation; TV: tricuspid valve; RA: right atrium.

Characteristic		MMI (n = 17)	TEE (n = 23)	*p*-Value
Device success *		100% (17/17)	100% (23/23)	1.000
TR reduction	1-grade	94% (16/17)	91% (21/23)	1.000
	≥2 grades	65% (11/17)	52% (12/23)	1.000
Mean no. clips/patient		1.5 ± 0.6	1.5 ± 0.5	0.851
Procedural time (min) **		113.6 ± 72.2	110.7 ± 54.9	0.888
Device time (min) ***		66.1 ± 35.1	58.7 ± 27.5	0.459
Fluoroscopy time (min)		14.4 ± 8.8	13 ± 7.1	0.578
Radiation dose (cGy)		4074.6 ± 2491.7	5125.1 ± 3827.6	0.330
TV mean gradient (mmHg)		1.8 ± 0.6	1.8 ± 0.7	0.896
RA pressure decrease (∆)		2.7 ± 1.3	3.2 ± 2.6	0.432
Length of hospital stay (days)		5.1 ± 3.2	8 ± 4.9	0.045
Technique	Bicuspidalization	18% (3/17)	30% (7/23)	0.719
	Clover	35% (6/17)	18% (4/23)	0.480
	“1-Clip” technique	47% (8/17)	52% (12/23)	1.000
Clip position	ant.-sept.	52% (14/27)	59% (20/34)	0.831
	post.-sept.	48% (13/27)	38% (13/34)	0.645
	ant.-post.	0%	3% (1/34)	1.000

**Table 4:** Adverse events (periprocedural to 12 months). * macrohematuria ** known intestinal angiodysplasia *** false aneurysm of arterial monitoring site **** mostly traumatic causes ***** two TEE probe related thermal lesions, one bleeding at gastroesophageal junction.

Event	MMI (n = 15)	TEE (n = 19)	*p*-Value
Cardiovascular mortality	1	2	1.000
All-cause mortality	2	4	0.661
Device related adverse events	0	0	---
Myocardial infarction	0	0	---
Major bleeding	1 *	1 **	1.000
Vascular complications	0	1 ***	1.000
Emergent cardiac surgery	0	0	---
New onset renal failure	0	0	---
New onset liver failure	0	0	---
Tricuspid valve stenosis	0	0	---
Stroke	0	0	---
Rehospitalization for AHF	4	8	0.734
Non-cardiac rehospitalization ****	7	13	0.575
Gastrointestinal bleeding	0	3 *****	0.237

**Table 5:** Efficacy secondary endpoints. Values are % (n) or mean ± SD (n). KCCQ: Kansas City Cardiomyopathy Questionnaire; NYHA: New York Heart Association; GFR: glomerular filtration rate; BUN: blood urea nitrogen; AST: aspartate aminotransferase; ALT: alanine transaminase; NT-pro BNP: N-terminal pro-B type natriuretic peptide.

Variable		MMI			TEE			MMI vs. TEE (∆)
		Baseline (n = 17)	12 Months (n = 15)	*p*-Value	Baseline (n = 23)	12 Months (n = 20)	*p*-Value	*p*-Value
Quality of Life								
KCCQ Score (pts.)		32.3 ± 18.7	61.9 ± 19.6	<0.001	26.7 ± 16.7	48.6 ± 20.9	<0.001	0.441
6-minute walk test (m)		183.2 ± 91.4	284.7 ± 114.7	<0.001	162.1 ± 94.1	247.8 ± 114.2	<0.001	0.541
NYHA class reduction	1-grade	--	15/15 (100%)	--	--	20/20 (100%)	--	1.000
	≥2 grades	--	5/15 (33%)	--	--	2/20 (10%)	--	0.112
Major organ systems								
GFR (mL/m^2^/1.73m^2^)		55.3 ± 15.9	59.4 ± 16.3	0.152	52.4 ± 18.4	55.2 ± 20.6	0.326	0.762
BUN (mg/dL)		56.1 ± 30.6	41.4 ± 15.3	0.014	62.7 ± 32.2	56.3 ± 26.1	0.181	0.244
AST (U/L)		34.2 ± 15.7	30.6 ± 11.8	0.085	33.4 ± 24.1	24.3 ± 8.3	0.033	0.274
ALT (U/L)		23.5 ± 18.5	20.5 ± 12.3	0.451	21 ± 14.6	16.8 ± 12.5	0.176	0.807
Bilirubin (mg/dL)		1.17 ± 0.97	0.90 ± 0.59	0.163	0.86 ± 0.84	0.69 ± 0.44	0.082	0.636
NTproBNP (pg/mL)		2594.5 ± 1756.6	2076.4 ± 1304.1	0.103	4103.5 ± 6018	3415.8 ± 5294	0.047	0.704

## Data Availability

The data presented in this study are available on request from the corresponding author due to restrictions imposed by the ethics committee.

## Supplementary Materials

The following supporting information can be downloaded at: https://www.mdpi.com/article/10.3390/jcm13102833/s1, Figure S1: TEE periprocedural protocol; Figure S2: TTE follow-up; Figure S3: Procedural steps of multimodality imaging guidance TR reduction to baseline; Figure S4: TR reduction to baseline; Table S1: TEE screening protocol; Table S2: Baseline characteristics (extended); Table S3: Echocardiographic parameters; Video S1: one-chamber TTE view; Video S2: TTE screening for the four right-sided chamber views; Video S3: procedural views of MMI-guided T-TEER.
