# Supplementary material for: Personalized Guidance of Edge-to-Edge Transcatheter Tricuspid Valve Repair by Multimodality Imaging
Source: J Clin Med. 2024 May 11;13(10):2833. doi: 10.3390/jcm13102833 (PMC11122187; doi:10.3390/jcm13102833)
Supplement: Supplementary file 1 [file jcm-13-02833-s001.zip › jcm-2967973-supplementary.pdf]

SUPPLEMENTARY MATERIAL

Supplementary Figure S1: TEE periprocedural protocol

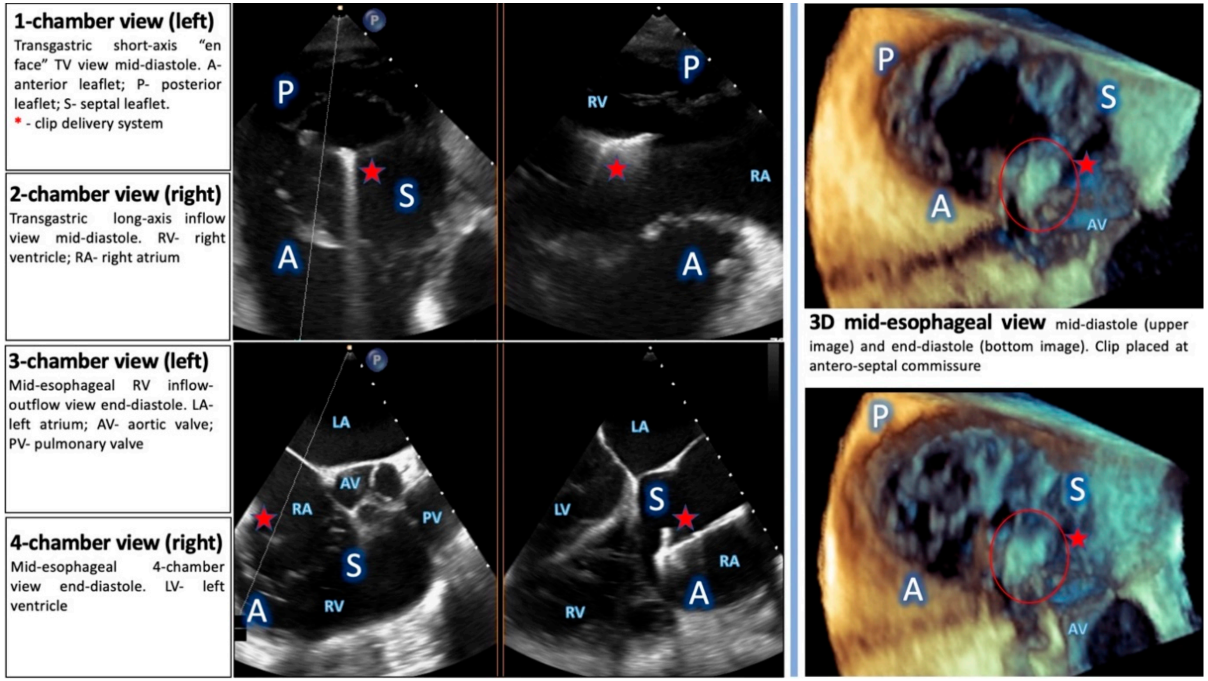

Typical periprocedural TEE (transesophageal echocardiography) views during T-TEER, grouped according to the 4 right-sided chamber views. TV: tricuspid valve, RV: right ventricle, RA: right atrium, A: anterior leaflet, P: posterior leaflet, S: septal leaflet, AV: aortic valve, PV: pulmonary valve, LV: left ventricle, *clip delivery system

Supplementary Figure S2: TTE follow-up

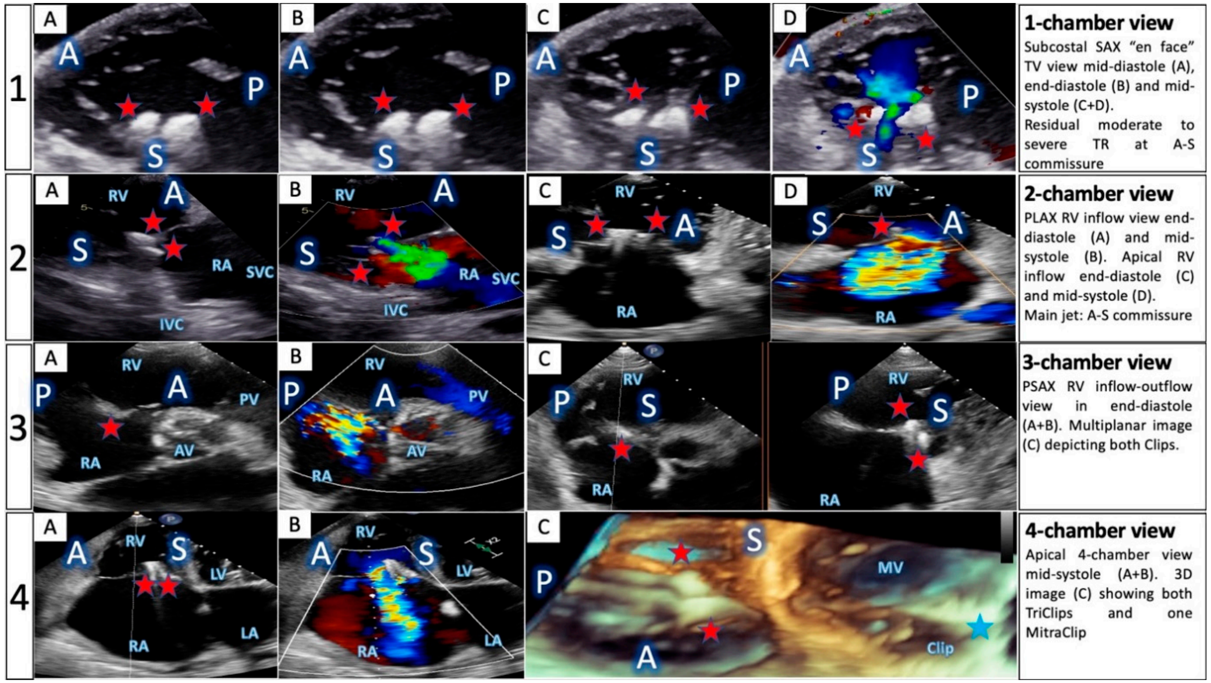

Follow-up investigation after T-TEER, based on the concept of the 4 right-sided chamber views in a patient with reduction from torrential to severe TR. A: anterior leaflet, P: posterior leaflet, S: septal leaflet, RV: right ventricle, RA: right atrium, IVC: inferior vena cava, SVC: superior vena cava, AV: aortic valve, PV: pulmonary valve, LV: left ventricle, LA: left atrium, *2xTriClip™, *MitraClip™.

Supplementary Figure S3. Procedural steps of multimodality imaging guidance

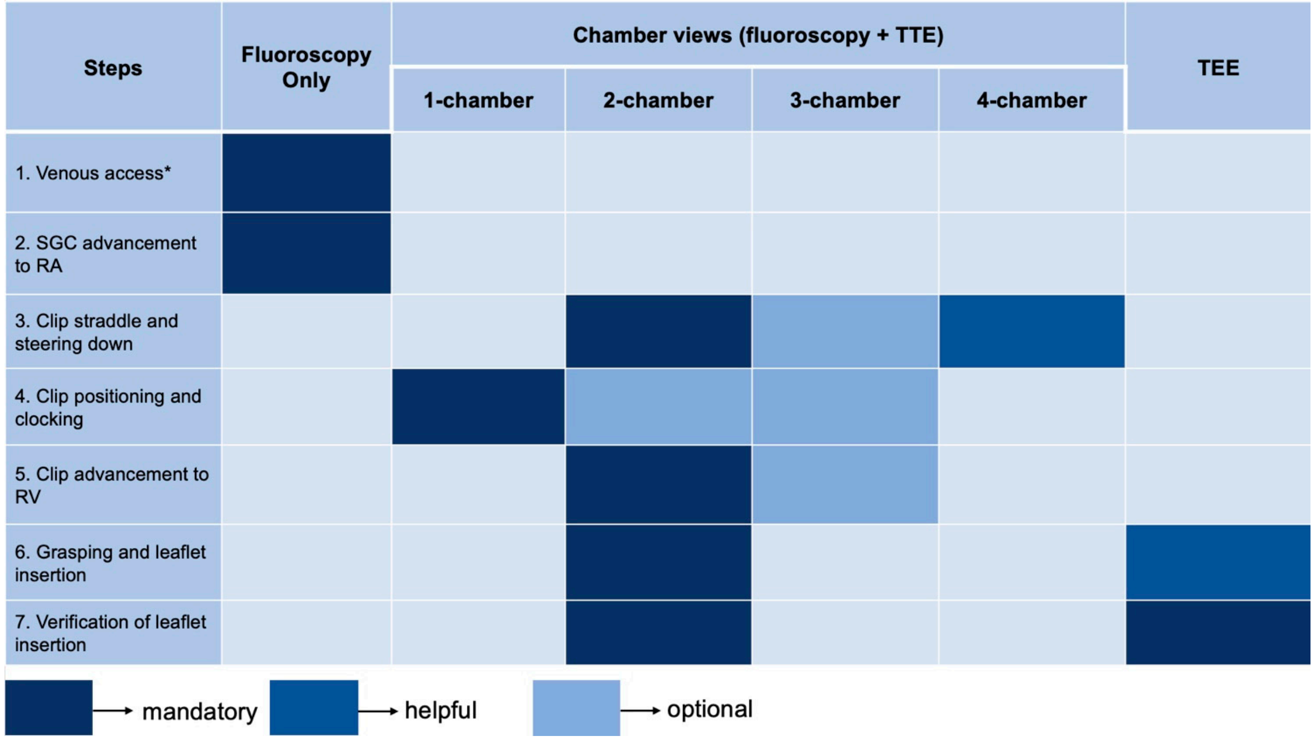

TTE: transthoracic echocardiography; TEE: transesophageal echocardiography; 1- to 4-chamber annotations refer to the 4 right-sided chamber views explained in the imaging and procedural protocols; SGC: steerable guide catheter; RA: right atrium; RV: right ventricle; * ultrasound-guided puncture.

Supplementary Figure S4. TR reduction to baseline

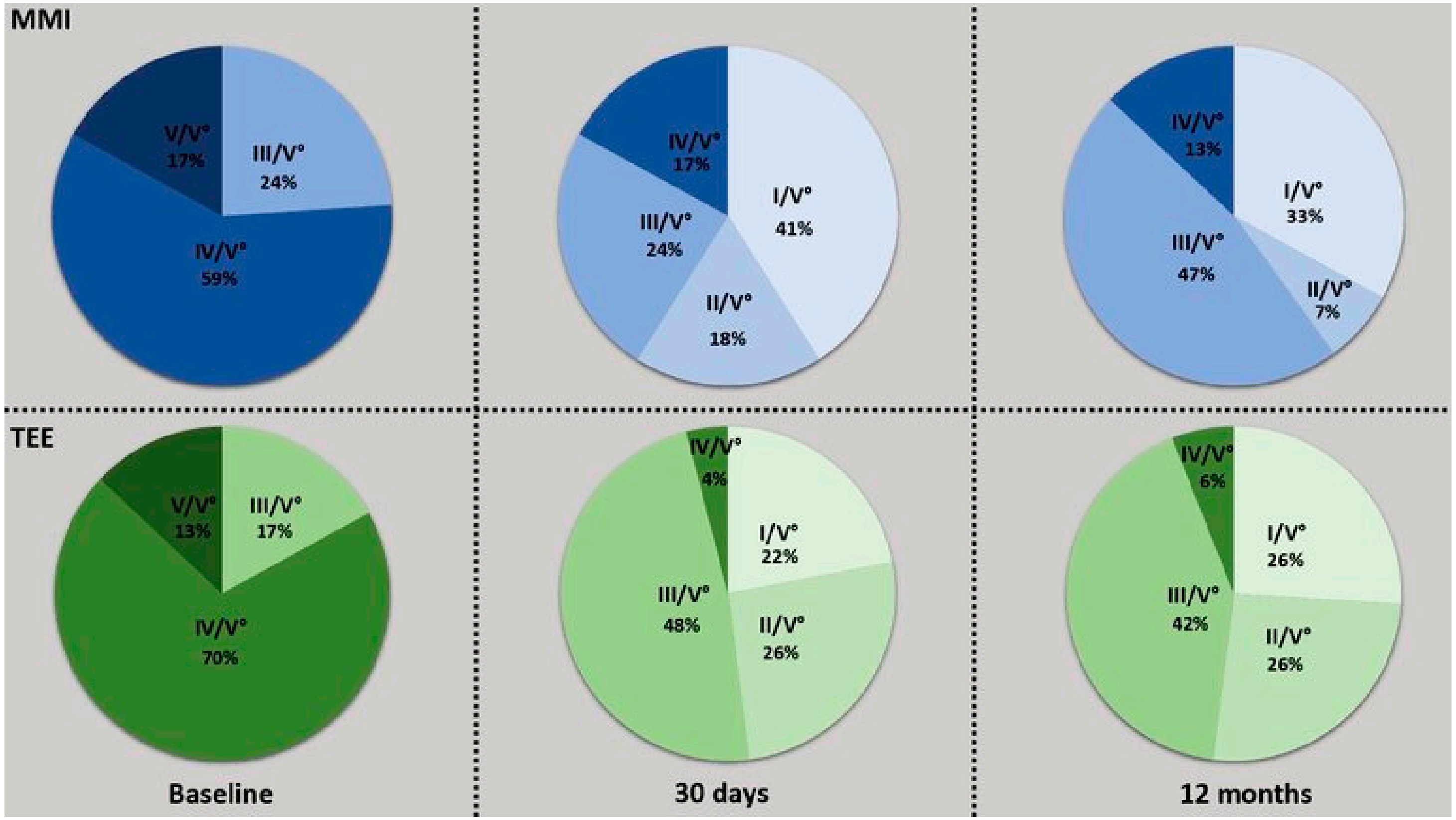

Comparison of regurgitation reduction between the multimodality (MMI) and transesophageal echocardiography (TEE) groups, according to the 5-grade classification (I/V° to V/V°) for tricuspid regurgitation (TR).

Supplementary Table S1: TEE periprocedural protocol

**TEE View**		**Focus**	
		**Morphology**	**Guiding**
Bicaval view		TV position related to caval veins	Orientation SGC towards TV
Transgastric views	Short axis (**RH 1 chamber view**)	"en face" TV view	Selecting clip target
	Long axis (**RH 2 chamber view**)	RV inflow	Clip orientation / perpendicularity to TR jet
Mid-esophageal views	0° (**RH 4 chamber view**)	SL length, tethering, restriction	SL grasping
	40-90° (**RH 3 chamber view**)	SL visualization<br>AL vs PL distinction<br>Leaflet length, tethering, restriction	Grasping all leaflets<br>Coaptation gap
	90-130°	SL visualization<br>AL vs PL distinction<br>Leaflet length, tethering, restriction	Grasping all leaflets<br>Coaptation gap
3D-views		Leaflets and commissures<br>CIED leads	Clip orientation

TEE: transesophageal echocardiography, RH: right heart, TV: tricuspid valve, RV: right ventricle, SGC: steering guide catheter, AL: anterior leaflet, PL: posterior leaflet, SL: septal leaflet

Supplementary Table S2. Baseline characteristics (extended)

Characteristic		*MMI (n=17)*	*TEE (n=23)*	*p-value*
Clinical				
Age (years)		83.1±4.1	81±5.3	0.182
Female sex		10 (59%)	10 (44%)	0.595
TTE acoustic window	Excellent	17 (100%)	0 (0%)	**<0.001**
	Good	0 (0%)	2 (9%)	0.506
	Moderate	0 (0%)	13 (57%)	0.004
	Poor	0 (0%)	8 (34%)	0.037
BMI (kg/m^2)		22.9±1.1	30.4±3.7	**<0.001**
EuroSCORE II (%)		10.1±8.2	8.6±5.6	0.496
Frailty (indices)		2.5±0.9	2.2±1.1	0.890
STS Score MVR (%)		11.1±7.4	10.6±5.9	0.813
STS Score MVr (%)		8.7±7.7	7.8±5.8	0.675
Major organ compromise		2.5±1.4	2.3±1.1	0.616
NYHA class III-IV		15 (88%)	20 (87%)	1.000
RHF hospitalizations		2.8±0.7	2.5±0.7	0.188
KCCQ Score		32.3±18.7	26.7±14.7	0.295
6-minute walk test		183.2±91.3	162.1±94.1	0.482

Characteristic		*MMI (n=17)*	*TEE (n=23)*	*p-value*
Comorbidities				
Atrial fibrillation		16 (94%)	23 (100%)	1.000
Pulmonary hypertension		16 (94%)	22 (96%)	1.000
Type 2 diabetes		6 (35%)	10 (43%)	0.773
Arterial hypertension		17 (100%)	23 (100%)	1.000
Hyperlipoproteinemia		6 (35%)	15 (65%)	0.405
Tobacco		2 (12%)	2 (9%)	1.000
COPD		3 (18%)	6 (26%)	0.719
CKD stage 3-5		14 (82%)	19 (83%)	1.000
Prior stroke / TIA		3 (18%)	5 (22%)	1.000
Coronary artery disease		8 (47%)	15 (65%)	0.339
Coronary bypass graft		1 (6%)	2 (9%)	1.000
Prior myocardial infarction		1 (6%)	5 (22%)	0.380
Peripheral artery disease		4 (23%)	7 (30%)	1.000
Pacemaker / ICD / CRT		5 (29%)	4 (17%)	0.712
Prior MV repair	percutaneous	7 (41%)	3 (13%)	0.164
	surgical	1 (10%)	1 (4%)	1.000

Data presented as n (%) or mean ± SD. BMI : body mass index ; EuroSCORE: European System for Cardiac Operative Risk Evaluation Score; STS: Society of Thoracic Surgeons predicted risk of mortality -> calculated based on either isolated mitral valve replacement (MVR) or mitral valve repair (MVr); NYHA: New York Heart Association; RHF: right heart failure; KCCQ: Kansas City Cardiomyopathy Questionnaire; COPD: chronic obstructive pulmonary disease; CKD: chronic kidney disease; TIA: transient ischemic attack; ICD: implantable cardioverter defibrillator; CRT: cardiac resynchronization therapy; MV : mitral valve

Supplementary Table S3. Echocardiographic parameters

Variable	*TTE*			*TEE*			*TTE vs TEE (Δ)*
	Baseline (n=17)	*12 months (n=15)*	*p-value*	*Baseline (n=23)*	*12 months (n=19)*	*p-value*	*p-value*
TR parameters							
PISA EROA (mm²)	82.1±34.5	33.7±35.7	<0.001	77.5±36	23.8±20.1	<0.001	0.764
Mean vena contracta (mm)	16.2±4.8	7.8±5.5	<0.001	16.8±4.3	6.2±3.2	<0.001	0.542
TR volume (mL/beat)	70.4±22.7	31.8±34.4	<0.001	84.2±36.5	24.2±19.8	<0.001	0.112
PISA (mm)	9.1±1.7	5.9±4.3	0.006	9.5±2.3	5.3±2.8	<0.001	0.940
Jet area (4ch) (cm²)	18.8±6.7	10.8±9.4	<0.001	17.3±7.5	7.8±4.7	<0.001	0.517
IVC diameter (mm)	24.1±7.7	15.7±7.5	<0.001	23.9±5.8	17.7±6.6	<0.001	0.639
Hepatic vein flow reversal (%)	13 (77%)	4 (27%)	0.120	19 (83%)	1 (5%)	0.001	0.212
Tenting height (mm)	8.5±1.8	--	--	8.5±1.9	--	--	0.492
Coaptation gap	7.7±2.7	--	--	8.1±2.4	--	--	0.325
Right heart remodeling							
RA indexed volume (mL/m²)	99.3±61.9	58.4±39.9	<0.001	86.1±33.7	39.1±30.4	<0.001	0.882
Base RVEDD (4ch) (mm)	52.1±9.1	48.9±9.3	0.001	55.5±6.8	49.6±7.4	<0.001	0.464
TV annular diameter (mm)	44.1±7.7	41.1±8.7	<0.001	46.1±6.5	41.6±7.1	<0.001	0.101
Ventricular systolic function							

RV TAPSE (mm)	16.1±4.1	20.6±5.2	<0.001	15.7±3.8	19.3±3.4	<0.001	0.702
RV FAC (%)	33.5±7.2	41.8±10	0.023	29.5±8.7	33.3±15.3	0.029	0.469
Annular velocity TDI (cm/s)	9.2±3.2	13.7±4.3	0.023	8.7±1.9	17.3±11.4	0.002	0.729
LV ejection fraction (%)	51.5±12.4	52.6±8.9	0.095	53.9±12.9	54.1±9.9	0.054	0.628
Hemodynamic parameters							
TR peak velocity (cm/s)	311.9±46.9	282.3±33.7	0.008	331.2±40.1	287.6±54.2	<0.001	0.692
PAPs	58±11.7	48.3±7.3	0.015	62.8±10.6	48.4±14.8	<0.001	0.718
LV stroke volume (mL)	54.8±10.6	60.1±9.7	<0.001	54.2±8.8	56.7±10	0.300	0.410
Cardiac output (L/min)	3.9±0.9	4.4±0.7	0.002	3.8±0.9	3.9±0.7	0.501	0.108
RV stroke volume (mL)	60.5±10.9	65.4±10.4	0.001	59.1±10.8	62.2±11.2	0.013	0.261

Data presented as n (%) or mean ± SD. PISA EROA: proximal isovelocity surface area effective regurgitant orifice area; TR: tricuspid regurgitation; ch: chamber; IVC : inferior vena cava ; RA: right atrium; RVEDD: right ventricular end-diastolic diameter; TV: tricuspid valve; TAPSE: tricuspid annular plane systolic excursion; FAC: fractional area change; TDI: tissue doppler imaging; LV: left ventricle; PAPs: pulmonary systolic artery pressure

VIDEO LEGENDS

Video S1. One-chamber TTE view

Video illustration of the 1-chamber right heart view in a patient with massive tricuspid regurgitation (Figure 1), containing the 2D subcostal short axis and the 3D "en face" tricuspid valve visualization derived from subcostal, apical or parasternal views.

Video S2. TTE screening for the 4 right-sided chamber views

Video compilation matching Figure 1 (Main text)

Video S3. Procedural steps of MMI guided T-TEER

Video sequence matching Figure 4 (Main text)
